# Comprehensive genome-wide analysis for the safety assessment of microbial biostimulants in agricultural applications

**DOI:** 10.1099/mgen.0.001391

**Published:** 2025-04-28

**Authors:** Gabriele Bellotti, Claudia Cortimiglia, Maria Elena Antinori, Pier Sandro Cocconcelli, Edoardo Puglisi

**Affiliations:** 1 Department for Sustainable Food Process, Università Cattolica del Sacro Cuore, Piacenza, Italy

**Keywords:** antimicrobial resistance, horizontal gene transfer (HGT), plant growth-promoting rhizobacteria (PGPR), risk analysis, safety evaluation, sustainable agriculture, virulence factors

## Abstract

Microbial biostimulants (MBs) offer a sustainable approach to agriculture by helping to reduce reliance on synthetic fertilizers. However, as MBs are intentionally released into the environment, their safety should be rigorously assessed. While taxa with qualified presumption of safety (QPS) benefit from established safety indications, non-QPS taxa lack such guidance. To address this gap, we propose a pipeline combining whole genome sequencing (WGS) and extensive literature search (ELS) data to evaluate microbial safety. We analysed public genomes of three QPS species (*Rhodopseudomonas palustris*, *Bacillus velezensis*, *Priestia megaterium*) and four non-QPS genera (*Arthrobacter*, *Azotobacter*, *Azospirillum*, *Herbaspirillum*), screening them for virulence factors (VFs), antimicrobial resistance (AMR) genes and mobile genetic elements (MGEs). Results confirmed the safety of QPS taxa, revealing no VFs and only a few intrinsic and non-clinically relevant AMRs. Among non-QPS taxa, VF hits were more prevalent in *Azotobacter* and *Azospirillum* spp., though they were mostly related to beneficial plant interactions rather than pathogenicity. AMR genes in non-QPS taxa were primarily associated with efflux pumps or were sporadically distributed. Notably, the only genus-wide pattern observed was that most *Azospirillum* and *Herbaspirillum* genomes harboured chromosomally encoded *β*-lactamases sharing similar genetic structures; however, the detected *β*-lactamase (*bla*) genes were distantly related to clinically relevant *bla* variants, and the absence of MGEs suggests a low risk of horizontal gene transfer, indicating the overall safety of these genera. In general, this WGS–ELS framework provides a robust tool for assessing the safety of non-QPS MBs, supporting regulatory decision-making and ensuring their safe use in sustainable agriculture while safeguarding public health.

**Impact Statement**

The implications of the safety evaluations conducted in this study are of interest to the agricultural industry, particularly in terms of regulatory approval, market acceptance and practical application. The workflow used to achieve the discussed results encourages the use of whole genome sequencing for the risk assessment of microbial biostimulants. This study underscores the importance of integrating genomic and literature-based approaches to establish a comprehensive safety profile for microbial biostimulants. By demonstrating that qualified presumption of safety (QPS) taxa exhibit low-risk profiles and identifying specific antimicrobial resistance (AMR) and virulence factor genes within non-QPS taxa, the research provides valuable insights for regulatory frameworks and industry practices. Ensuring the safe application of microbial biostimulants in agriculture not only promotes sustainable farming practices but also mitigates potential public health risks associated with AMR.

## Data Summary

All genome data used in this study were retrieved from the NCBI GenBank repository as of July 2024. This includes 400 genomes of *Priestia megaterium* (formerly *Bacillus megaterium*), 890 genomes of *Bacillus velezensis* (formerly *Bacillus amyloliquefaciens* subsp. *plantarum*) and 35 genomes of *Rhodopseudomonas palustris* for QPS taxa, along with 31 genomes of *Azotobacter* spp*.*, 88 genomes of *Azospirillum* spp*.*, 47 genomes of *Herbaspirillum* spp*.* and 200 genomes of *Arthrobacter* spp. for non-QPS taxa. Accession numbers for all genomes are provided in the supplementary material (Table S1). Relevant antimicrobial resistance and virulence factor genes were identified using the comprehensive antibiotic resistance database, ResFinder and the virulence factor database. Mobile genetic elements were evaluated with ICEfinder. The authors confirm all supporting data, code and protocols have been provided within the article or through supplementary data files.

## Introduction

The role of microbes in promoting plant growth has a rich and longstanding history [1,2]. Recent advances in science and technology have greatly enhanced our understanding of complex ecosystems such as soil, highlighting the role of microbial communities in improving the availability of nutrients and regulating phytohormones, positively affecting plant life [3,5]. The rhizosphere has been shown to be a source of micro-organisms with beneficial properties [6]. Fungi and bacteria isolated from this niche have been studied and their valuable functions exploited to increase plant production [7]. As a result, the development of microbial biostimulants (MBs) has increased over the past decades, aiming to reduce our global reliance on synthetic fertilizers [8]. While MBs naturally interact with target crops and are generally considered ecologically sustainable, their large-scale use presents potential risks [9].

Soil is one of the most biologically diverse ecosystems, harbouring a vast array of microbial species that constantly interact with each other and the surrounding environment [10]. This diversity includes the presence of naturally occurring opportunistic pathogens [11,12], and among the numerous interactions that occur in soil, horizontal gene transfer (HGT) events facilitate the exchange of genetic material between different species, including genes related to antimicrobial resistance (AMR) or virulence factors (VFs). These events, although little studied in soil, may lead opportunistic pathogens to acquire genes that enhance their resilience and virulence [13,15]. For this reason, the use of MBs should include preliminary risk assessment analysis to ascertain the absence of genes of concern that could then be propagated within the soil. This risk assessment of MBs is crucial, also considering that their introduction into crops inevitably leads to their entry into the animal and human food supply [11,16], potentially amplifying the spread of AMR or VF genes throughout the food chain, and representing a risk for humans.

In 2022, the European Biostimulants Industry Council (EBIC) issued a position paper [17] advocating for expanded research and innovation in MBs beyond the microbial species currently regulated under Regulation (EU) 2019/1009, which includes the bacterial genera *Azospirillum*, *Azotobacter* and *Rhizobium*, along with mycorrhizal fungi. The EBIC paper highlights several promising microbial species identified in the scientific literature but emphasizes the significant gaps in knowledge regarding their safe use, underscoring the need for further research and harmonized safety regulations. Although Regulation (EU) 2019/1009 proposes a list of species that may be considered MBs, it would be good practice to assess their risk, also considering that the taxonomy of these microbial genera is not yet adequately defined. While a risk assessment framework for MBs has yet to be defined, an approach based on the qualified presumption of safety (QPS) concept may represent the starting point, given that MBs ultimately enter the food chain and may impact the human gut. The QPS framework has been introduced by the European Food Safety Authority (EFSA) to assess the safety of biological agents in food and feed. The QPS framework classifies certain microbes as presumably safe, based on four criteria: (i) clear taxonomic identification, (ii) sufficient scientific and historical knowledge, (iii) absence of safety concerns like pathogenicity or AMRs and (iv) intended end use. While the QPS system reduces regulatory burdens for companies developing products from QPS-approved species, it relies heavily on pre-existing knowledge of the species in question. This reliance on historical data limits the application of QPS for MBs, which have a much shorter history of use compared to fermented foods or probiotics [18].

**Fig. 1.**
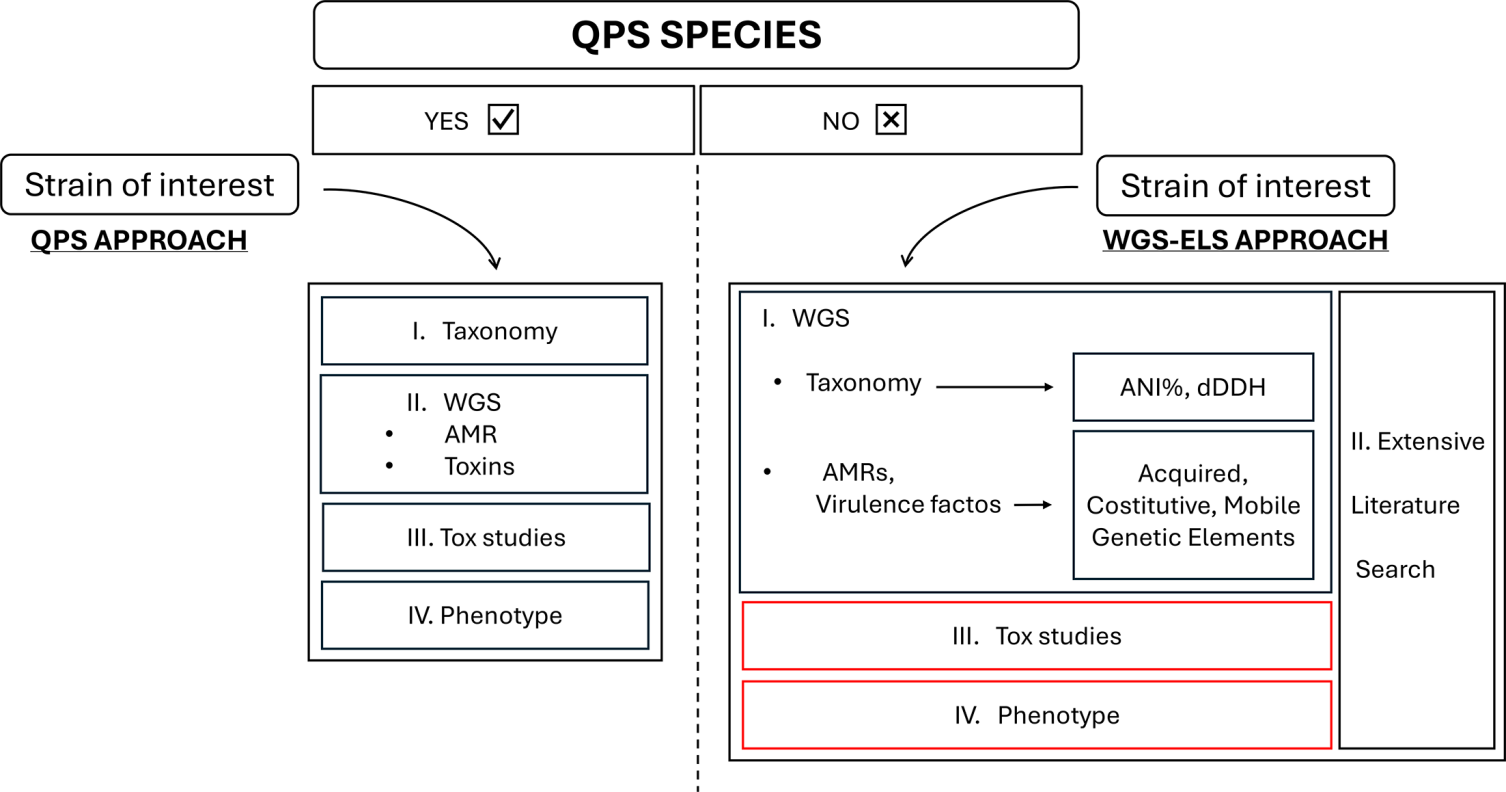
Framework comparison of the four main steps used to assign the QPS status to microbial strains (left) and the WGS–ELS approach for microbial safety assessment. Two customizable steps (highlighted in red boxes) allow for adjustments based on the genomic analysis findings, such as lowering detection thresholds or expanding database searches, to ensure comprehensive identification of important safety aspects.

To shed light on MB safety, we propose the application of a whole genome sequencing (WGS) approach that mirrors the QPS framework to identify the taxonomy and gather critical safety data, namely AMR genes, VF genes and mobile genetic elements. This is followed by a comprehensive literature review using extensive literature search (ELS) tools to collect relevant scientific knowledge. WGS has been successfully used to characterize the genetic content of microbial strains for safety purposes in both food [19] and agricultural sectors [20], and it can offer a rigorous yet efficient assessment also for non-QPS MBs. Thus, we applied a genome-wide analysis to publicly available bacterial genomes of species commonly known as MBs. We selected *Rhodopseudomonas palustris*, *Bacillus velezensis* and *Priestia megaterium*, which are recognized under the QPS framework, due to their established safety profile and widespread use in the food and feed and agricultural sector [21]. These species have been extensively studied for their plant growth-promoting traits, including nitrogen fixation, phosphate solubilization and secondary metabolite production, making them key candidates in sustainable agricultural applications. In addition to these QPS species, we included four non-QPS genera to capture a broader spectrum of beneficial plant-associated bacteria. *Azotobacter* spp. (class *Gammaproteobacteria*) were chosen as free-living nitrogen-fixing bacteria known for their contribution to soil fertility and plant growth promotion [8]. *Azospirillum* spp. (class *Alphaproteobacteria*) were selected due to their well-documented association with plant roots, where they perform nitrogen fixation, exopolysaccharide production and auxin biosynthesis, improving root architecture [22]. *Herbaspirillum* spp. (class *Betaproteobacteria*) were included as representative plant endophytes with diazotrophic capabilities, known to promote plant growth by facilitating nutrient acquisition and producing phytohormones [23]. Finally, *Arthrobacter* spp. (class *Actinobacteria*) were incorporated due to their ability to colonize the rhizosphere and enhance plant growth through phosphate solubilization and the synthesis of plant-beneficial compounds [24]. By conducting this comprehensive analysis, we aim to provide a framework for using WGS in the safety assessment of non-QPS MBs. To visualize the potential impact of our integrated strategy, Fig. 1 presents a schematic of the workflow designed to integrate WGS and ELS in the approval process for beneficial agricultural microbes.

## Methods

### Selection of bacterial genomes and genome-relatedness

For QPS taxa, all publicly available genomes were retrieved from the Nation Center for Biotechnology Information (NCBI) GenBank repository in July 2024. This included 35 genomes of *R. palustris*, 400 genomes of *P. megaterium* (formerly *Bacillus megaterium*) and 890 genomes of *B. velezensis* (formerly *Bacillus amyloliquefaciens* subsp. *plantarum*). For each non-QPS genome, the officially recognized species were searched on the website ‘List of Prokaryotic names with Standing in Nomenclature’ (https://lpsn.dsmz.de/). All available genomes for each species were collected, resulting in 200 genomes for *Arthrobacter* spp., 31 genomes for *Azotobacter* spp., 88 for *Azospirillum* spp. and 47 for *Herbaspirillum* spp., retrieved from the NCBI GenBank repository in July 2024 as well. The complete list of the genomes used, including their accession number, is provided in Table S1, available in the online Supplementary Material.

Genome-relatedness was verified by assessing the genome-to-genome distance, specifically by calculating the average nucleotide identity (ANI) between each species and its respective type strain. A threshold above 95% was set to define species-level similarity, while an ANI of 85% was used for genus-level classification [25]. ANI analysis was performed using the FastANI algorithm v1.34 [26]. Genomes that fell below the 95% ANI threshold were further analysed using digital DNA–DNA hybridization (dDDH) through the type strain genome server at https://tygs.dsmz.de/ [27], with a dDDH threshold>70% to confirm species delineation [28]. Genomes showing ANI or dDDH values below these thresholds were re-examined against other type strains within the genus to determine the need for reclassification. Duplicate genomes, or those flagged as too short or problematic in NCBI, were excluded (Table S1).

To further validate the genome-relatedness results, phylogenetic trees were constructed for each non-QPS species using unique genomes. Phylogenetic analysis allows one to understand the relationships among different strains, which is essential for predicting the presence of genetic elements that may pose safety concerns [29]. By elucidating these relationships, we can identify clades or groups that may share specific genetic traits relevant to safety, thereby enhancing our ability to assess risks associated with the use of these microbes as biostimulants. Phylogenetic trees were generated using the ‘Bacterial genome tree’ service provided by BV-BRC (v3.39.10) described in [30]. Briefly, the phylogenetic trees were constructed using the alignment of the highest possible number of single-copy genes shared within each non-QPS genus. To ensure a strict selection, we applied a filtering criterion allowing zero deletions and zero duplications, making the alignment highly restrictive. Both amino acids and nucleotide sequences of those genes were aligned using MUSCLE [31] and the Codon_align function of biopython [32], respectively. The alignments were submitted to RaxML (v8.2.12) to build phylogenetic trees with 100 bootstrap iterations. The resulting Newick files were uploaded to the Interactive Tree of Life (iTOL) [33] to generate midpoint-rooted phylogenetic trees with bootstrap support values for each non-QPS genus.

### Relevant criteria for safety evaluation by ELS

Once the taxonomic classification was confirmed, the safety of each non-QPS taxon was assessed by searching for known safety concerns and relevant literature. The ELS method, using terms recommended by EFSA-BIOHAZ [34], was employed to locate case studies and reports of pathogenicity, as well as previously characterized AMRs or VFs. The search terms included the following combinations of terms: (‘Arthrobacter’ OR ‘Arthrobacter *’ OR ‘Azospirillum’ OR ‘Azospirillum *’ OR ‘Azotobacter’ OR ‘Azotobacter *’ OR ‘Herbaspirillum’ OR ‘Herbaspirillum *’) AND (‘antimicrobial resistan*’ OR ‘antibiotic resistan*’ OR ‘antimicrobial susceptibil*’) AND (‘toxin*’ OR ‘disease*’ OR ‘infection*’ OR ‘clinical*’ OR ‘virulen*’ OR ‘endocarditis’). The resulting list of articles was thoroughly reviewed to identify relevant studies, which helped in determining the existing scientific knowledge about these non-QPS genera.

### Detection of antibiotic resistance genes, VFs and toxins

To assess the presence of VFs, pathogenesis-related genetic markers (e.g. toxins, invasion and adhesion factors) were identified using the virulence factor database [35]. AMR gene detection was performed by querying two curated databases, ResFinder [36] and comprehensive antibiotic resistance database (CARD) [37]. A minimum coverage threshold of 60% and nucleotide sequence identity of 60% were applied to screen for lesser-known genetic elements. Nevertheless, as recommended by EFSA, only query hits above the thresholds of 70% length of coverage and 80% sequence identity were considered robust hits [38]. The ABRicate tool (https://github.com/tseemann/abricate) was used for mass screenings into contigs. All bioinformatic results, including hits with≥60% identity and≥60% coverage, were recorded. Hits above the 70% identity and 80% coverage thresholds were reviewed for relevance. The main results were visualized using bubble plots generated with ggplot2 in R Studio v4.4.1 [39,40].

### Comparison of *Azospirillum* and *Herbaspirillum β*-lactamases sequences, conserved regions and detection of insertion sequences

Although *β*-lactamase activity is only known in the literature for the genus *Azospirillum*, genomic analyses conducted in this study have revealed the presence of *β*-lactamases in the genus *Herbaspirillum* as well. Therefore, the nucleotide and amino acid sequences of *β*-lactamases were retrieved and aligned to construct phylogenetic trees based on sequence similarity using the Gene/Protein Tree service in BV-BRC [41]. Phylogenetic analysis of *β*-lactamase genes included the *bla_LRA_*_-1_ sequence (CARD accession number ARO:3002482), previously described in [42], as well as other known clinically important *β*-lactamase *bla_CTX_*_-M-38_ (ARO:3001900), *bla_CTX_*_-M-15_ (ARO:3001878), *bla_CTX_*_-M-5_ (ARO:3001868) and *bla_CTX_*_-M-65_ (ARO:3001926) described in [43]. The resulting Newick files were visualized using iTOL v5 to generate midpoint-rooted trees. The genomic context of these *β*-lactamase genes, including surrounding regions, was assessed using the Compare Region Viewer in BV-BRC. Finally, genomes flagged as ‘complete’ were uploaded to ICEfinder on ICEberg v3.0 [44] and were checked for the presence of insertion sequences (IS) or integrated conjugative elements (ICEs).

### *In vitro* AMR assay

To verify the phenotype corresponding to the *in silico* AMR predictions, *in vitro* susceptibility tests were conducted on four *Azospirillum* strains from our collection: *Azospirillum argentinense* UC4320 and UC4321 (isolated from Mali), *Azospirillum brasilense* Sp7 (from Brazil) and *Azospirillum* sp. UWAZO (from Canada). These strains were selected due to their inclusion in our genomic analysis and their diverse geographical origins, encompassing isolates from Africa, South America and North America. This diversity allowed us to assess the consistency of AMR profiles across genetically distinct strains within the *Azospirillum* genus.

The antibiotics tested were chosen based on the ISO 10932:2010 guidelines, which provide a standardized method for determining minimum inhibitory concentrations (MICs) for lactic acid bacteria intended for use in food. While *Azospirillum* spp. is not a lactic acid bacteria, these guidelines were applied as they offer a recognized framework for evaluating the safety of micro-organisms introduced into food products with potential human exposure. The antibiotics selected from the ISO 10932:2010 were as follows: ampicillin (0.032–16 mg µl^−1^), chloramphenicol (0.125–64 mg µl^−1^), clindamycin (0.032–16 mg µl^−1^), erythromycin (0.016–8 mg µl^−1^), gentamicin (0.5–256 mg µl^−1^), kanamycin (2–1024 mg µl^−1^), streptomycin (0.5–256 mg µl^−1^) and tetracycline (0.125–64 mg µl^−1^).

Bacterial suspensions were prepared from fresh agar cultures and adjusted to a McFarland standard of 1.0. Each suspension was then diluted 1:500 in double-strength Mueller–Hinton broth. Antibiotic solutions were prepared from concentrated stocks and diluted with distilled water to achieve the appropriate concentrations in 96-well microtitre plates (Sarstedt, Germany). For each test, 100 µl of bacterial suspension was added to 100 µl of the antibiotic solution in the wells. The plates were incubated at 30 °C for 48 h. After incubation, OD at 600 nm (OD600) was measured using a microplate reader (Synergy HTX, Agilent BioTek, USA) to determine the MICs. All measurements were conducted in triplicate, with both positive and negative controls included. The negative control consisted of wells containing only the growth medium (Muller Hinton), without the test strain or antibiotic. The positive control included the test strain in the medium along with the solvent used to dissolve the antibiotic but without the antibiotic itself. *Lactobacillus plantarum* ATCC 14917 was used as a quality control strain with known resistances.

## Results

### Genome selection, taxonomic identification and phylogeny

Based on ANI and digital dDDH, the genomes originally assigned to the genera *Arthrobacter*, *Azotobacter*, *Azospirillum*, *Herbaspirillum*, *Priestia* and *Bacillus* were re-evaluated, leading to the reclassification of some genomes. Out of the 1,691 genomes analysed (1,325 from QPS taxa and 366 from non-QPS taxa), a few genomes were excluded due to quality issues reported by NCBI; others were individuated as duplicates and were removed. Within the *Azotobacter* genus, one low-quality genome (GCA_003251205.1) and three mutant genomes (GCA_036687375.1, GCA_036687365.1 and GCA_036687355.1), altered by clustered regularly interspaced short palindromic repeats (CRISPR) interference and later resequenced [45], were removed. For the *Azospirillum* genus, three genomes (GCA_003243305.1, GCA_003241095.1 and GCA_003241645.1) were removed due to similar quality concerns and seven genomes were duplicates (GCA_000237365.1, GCA_008274945.1, GCA_001315015.1, GCA_000404045.1, GCA_002027385.1, GCA_900177475.1 and GCA_017876055.1). Additionally, eight *As. brasilense* genomes were reassigned to different species based on ANI and dDDH values: (i) GCA_002245955.1 was reassigned to *Azospirillum tabaci*, (ii) GCA_007827795.1 and GCA_016622085.1 were reassigned to *Azospirillum aestuarii*, (iii) GCA_003349955.1 and GCA_016652835.1 were reassigned to *As. argentinense*, (iv) GCA_022023855.1, GCA_005222205.1 and GCA_007827915.1 were reclassified as *Azospirillum baldaniorum*. Seven other *Azospirillum* genomes could not be confidently assigned to any specific species and were classified as *Azospirillum* sp. (GCA_008365405.1, GCA_007828645.1, GCA_007827815.1, GCA_016622105.1, GCA_040500525.1, GCA_000283655.1 and GCA_900177515.1). For *Herbaspirillum*, one genome (GCA_004366165.1) was reassigned from *Herbaspirillum seropedicae* to *Herbaspirillum huttiense*, one was a duplicate (GCA_013180375.1) and one genome (GCA_009914615.1) was classified as *Herbaspirillum* sp. since it could not be assigned to any other known species (Table S1). For the genus *Arthrobacter*, 11 genomes were duplicates and were removed, while GCA_039535055.1 was reclassified from *Arthrobacter citreus* to *Arthrobacter gandavensis*, GCA_030803685.1 from *Arthrobacter globiformis* to *Arthrobacter ipis*, while GCA_039528575.1 was reassigned from *A. gandavensis* to *A. citreus*, and 46 were reassigned to *Arthrobacter* spp. (Table S1).

For QPS taxa, genome-relatedness analysis led to the exclusion of five *P. megaterium* genomes (GCA_008180335.1, GCA_034045135.1, GCA_008728615.2, GCA_008728615.1 and GCA_008728535.2), two *B. velezensis* genomes (GCA_024741885.1 and GCA_025758105.1) and 24 *R*. *palustris*. These excluded genomes exhibited low species demarcation scores based on ANI and dDDH values, which fell below the established threshold required for confident species assignment and were thus removed. The low ANI and dDDH scores indicate poor alignment with the expected reference genomes, suggesting incorrect species designation or low-quality genomic data. After these exclusions and taxonomic adjustments, the final dataset comprised 1,294 QPS and 340 non-QPS unique genomes (Table S1).

**Fig. 2.**
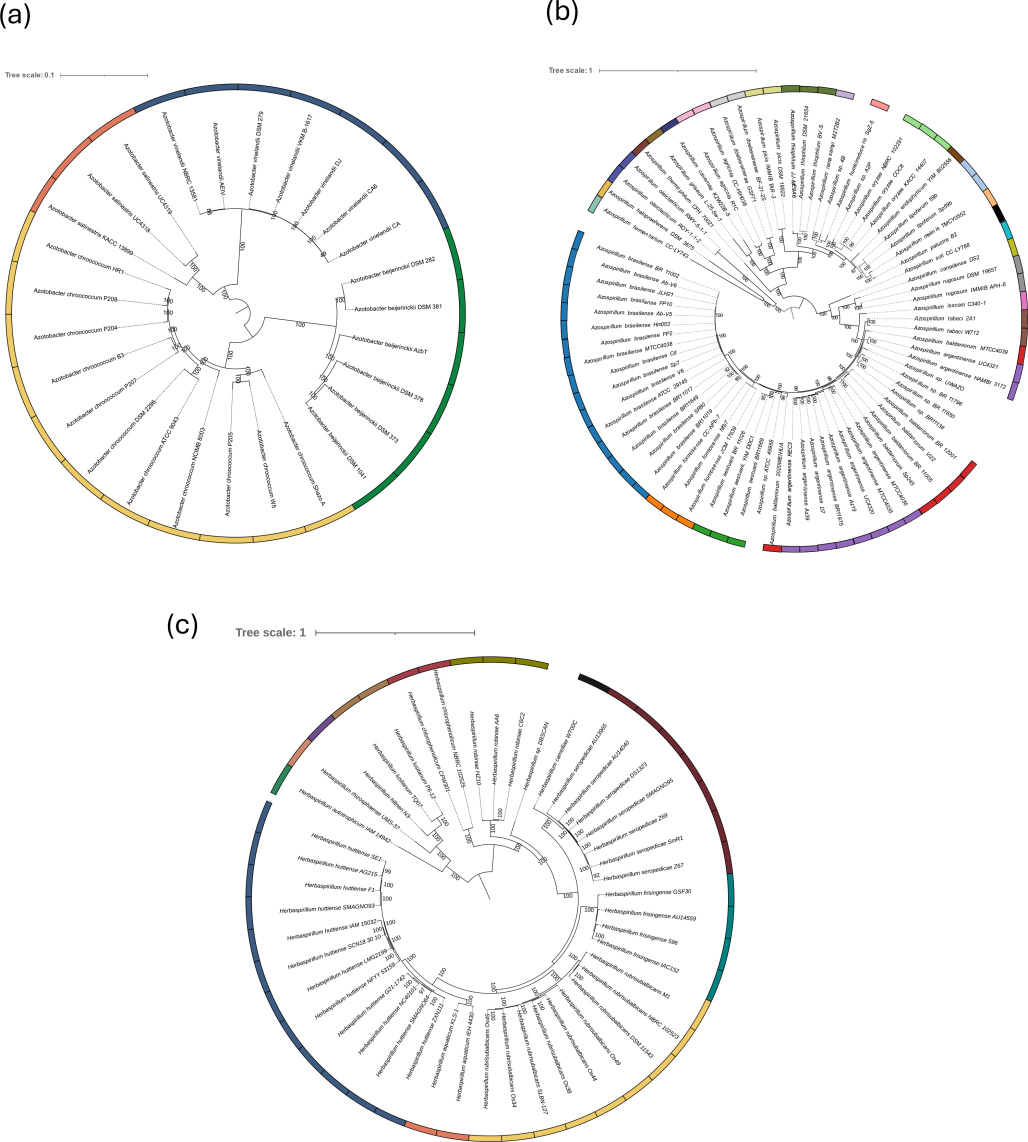
Phylogenetic classification of the 151 non-QPS bacterial genomes analysed based on their species-level phylogenetic relationships: (**a**) *Azotobacter* (27), (**b**) *Azospirillum* (78) and (**c**) *Herbaspirillum* (46). Colour separation indicates the clustering of genomes according to the species assigned following fastANI results. Numbers reported at the nodes are 1–100 bootstrap values.

In order to support the taxonomic assignment, phylogenetic trees were constructed by aligning single-copy core genes for each non-QPS taxa (Fig. 2). The alignment scores, statistical data and genes used to construct the phylogenetic trees are provided in Table S2. Phylogenetic analysis of the *Azotobacter* genus (Fig. 2a) revealed a clear distinction between the four species within the genus, confirming accurate species-level classification. Similarly, the *Herbaspirillum* genus displayed clear species delineation (Fig. 2c), consistent with modifications made according to its ANI results. In contrast, the phylogenetic analysis for *Azospirillum* (Fig. 2b) presented a more complex scenario. Despite several genomes being correctly assigned or reassigned to their respective type strains based on ANI and DDH values, the phylogenetic results revealed inconsistencies. The genomes of *As. baldaniorum* strains MTCC 4039 and 2020WEIHUA, as well as *As. argentinense* strains UC4321 and HAMBI 3172, were correctly classified at the species level using ANI and dDDH. However, they exhibited ambiguous clustering in the phylogenetic tree, with species appearing intermixed. For the genus *Arthrobacter*, the high genomic diversity posed additional challenges for phylogenetic reconstruction. The substantial variation among genomes allowed for an alignment based on only a few shared genes (Table S2), limiting the resolution of the phylogenetic analysis. Nevertheless, the phylogenetic results fully confirmed the classifications based on ANI and dDDH. The extensive genomic diversity within the genus was further underscored by the fact that 46 genomes were reclassified as *Arthrobacter* sp., as they neither reached the ANI or dDDH thresholds nor clustered with any reference strain in the phylogenetic analysis (Table S1 and Fig. S1).

### Genome-wide detection of VFs and AMRs

The list of hits exceeding the thresholds of 70% coverage and 80% identity for both QPS and non-QPS taxa is presented in Table S3. For QPS taxa, no VFs were identified in any of the 395 *P*. *megaterium* and 11 *R*. *palustris* genomes, while a single VF hit was identified among the 888 genomes of *B. velezensis*, specifically a phenazine biosynthesis gene (*phzA*1) in strain A6 (GCA_004118065.1).

AMR findings in *B. velezensis* included the widespread presence of *tetL* (in 80% of the genomes) and *clbA* (77%), where *tetL* confers resistance to tetracycline, and *clbA*, as part of the *cfr*-like resistance genes, confers resistance to multiple antibiotic classes, including phenicols and lincosamides. Additional AMRs, such as *bleO* (resistance to bleomycin) and *aadD* (conferring resistance to aminoglycosides, particularly kanamycin and neomycin), were found in a smaller proportion of the genomes (10%). In *P. megaterium*, the most prevalent AMR gene was *lsaB*, found in 25% of genomes, conferring resistance to clindamycin. Finally, a single AMR hit was found in *R. palustris* strain TIE-1 (GCA_000020445.1), specifically an aminoglycoside-3′-phosphotransferase (Table S3).

**Fig. 3.**
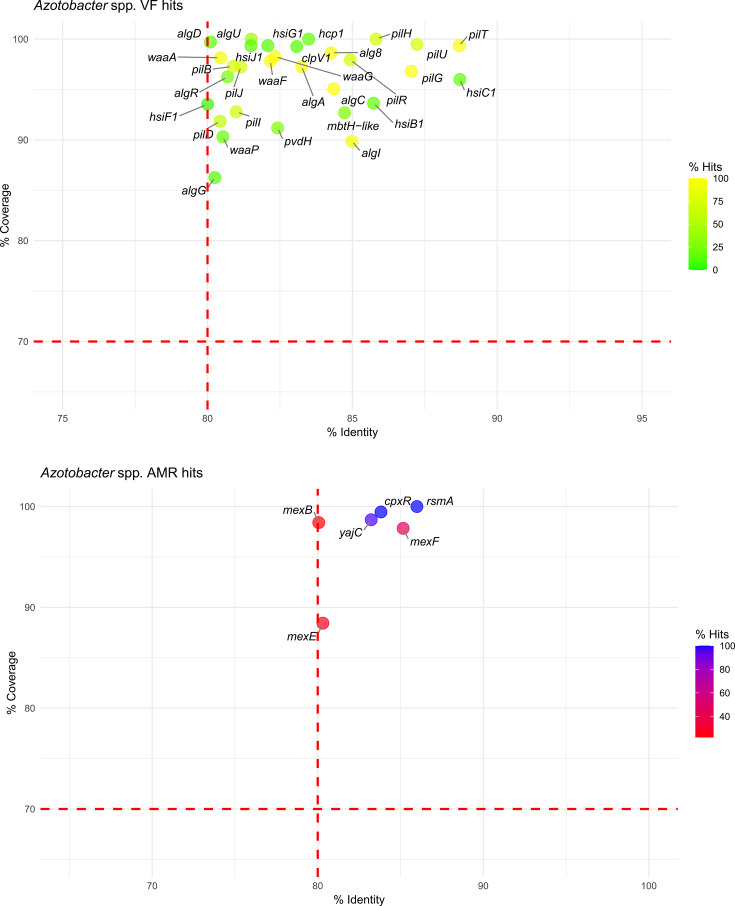
Bubble plot representing the VF (top) and AMR (bottom) hits in *Azotobacter* spp. The *X*-axis indicates the percentage identity, and the *Y*-axis shows the percentage coverage. Bubbles are coloured along a red-to-blue or yellow-to-green gradient based on the percentage of hits for AMRs or VFs, respectively. Red dashed lines represent the recommended thresholds at 80% identity and 70% coverage.

Different VF hits were detected in non-QPS taxa, particularly across the 27 *Azotobacter* genomes, where 30 relevant VFs were identified. Eight of these VF hits were linked to genes involved in alginate production, such as *algA*, *algC* and *alg8*, which are present in nearly all *Azotobacter* genomes. Similarly distributed, an additional eight hits were associated with type IV pili genes (*pil*), seven hits were related to type VI secretion system (T6SS) proteins (*hsiB*1, *hsiC*1, *hsiF*1) and four hits of lipopolysaccharide (LPS) genes (*waaA*, *waaF*, *waaG*, *waaP*). For AMRs, *Azotobacter* genomes exhibited six genetic features related to efflux pumps, including *mexB* (22% of genomes), *mexE* (25%) and *mexF* (44%), along with their regulatory genes *cpxR* (100%), *yajC* (96%) and *rsmA* (100%) (Fig. 3).

**Fig. 4.**
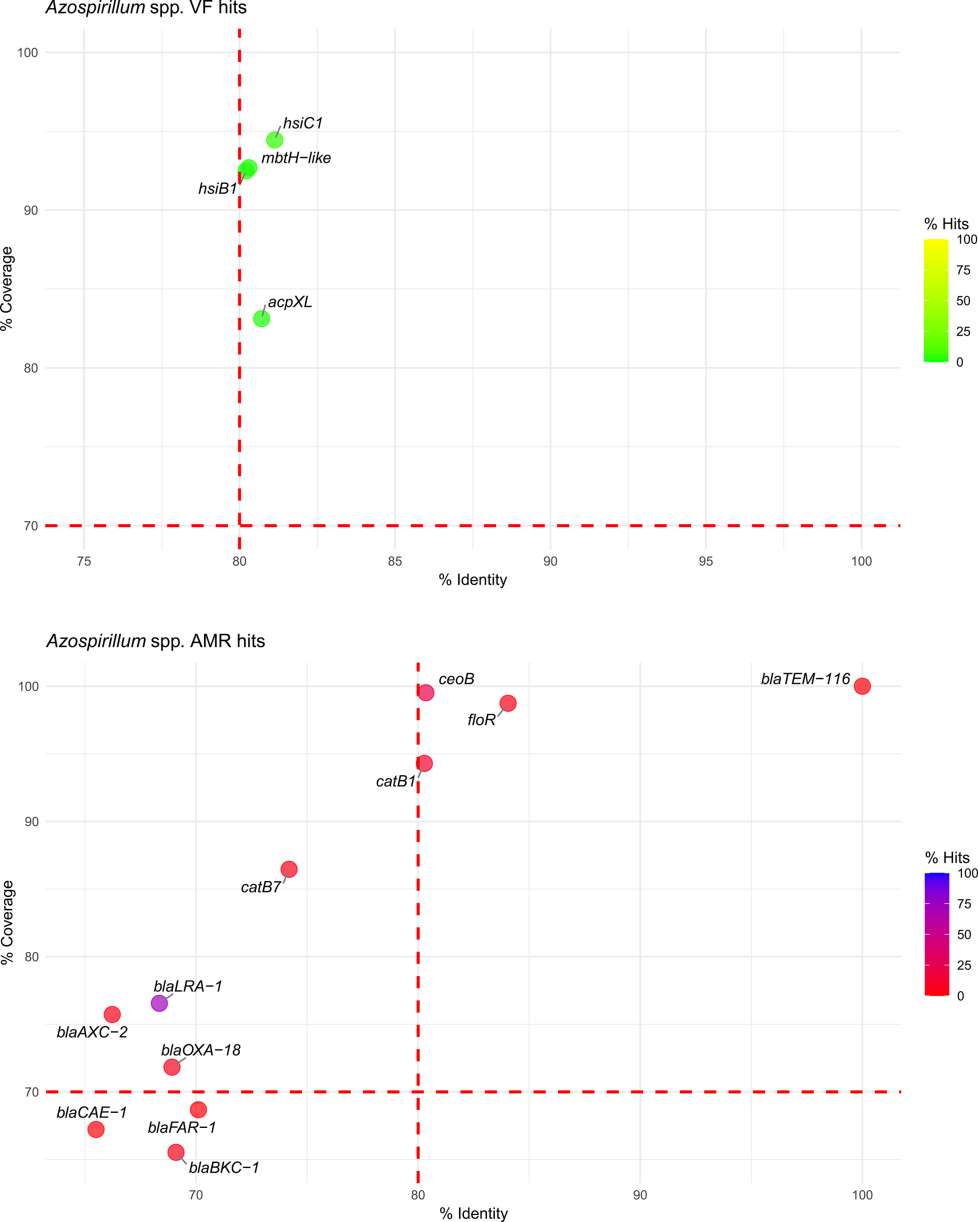
Bubble plot representing the VF (top) and AMR (bottom) hits in *Azospirillum* spp. The *X*-axis indicates the percentage identity, and the *Y*-axis shows the percentage coverage. Bubbles are coloured along a red-to-blue or yellow-to-green gradient based on the percentage of hits for AMRs or VFs, respectively. Red dashed lines represent the recommended thresholds at 80% identity and 70% coverage.

In *Azospirillum* spp., four VFs were identified across 78 genomes, with two hits associated with T6SS genes that are in fact reported as having a positive role in plant–microbe interaction [46]. The AMR profile revealed *ceoB* (22% of genomes), *catB*1 (10%) and *floR* (4%), showing a potential resistance to chloramphenicol antibiotics in some strains. A single broad-spectrum *β*-lactamase hit (*bla_TEM_*_-116_) was identified exclusively in *As. brasilense* strain Ab-V6 (GCA_002940755.1) (Fig. 4 and Table S3).

**Fig. 5.**
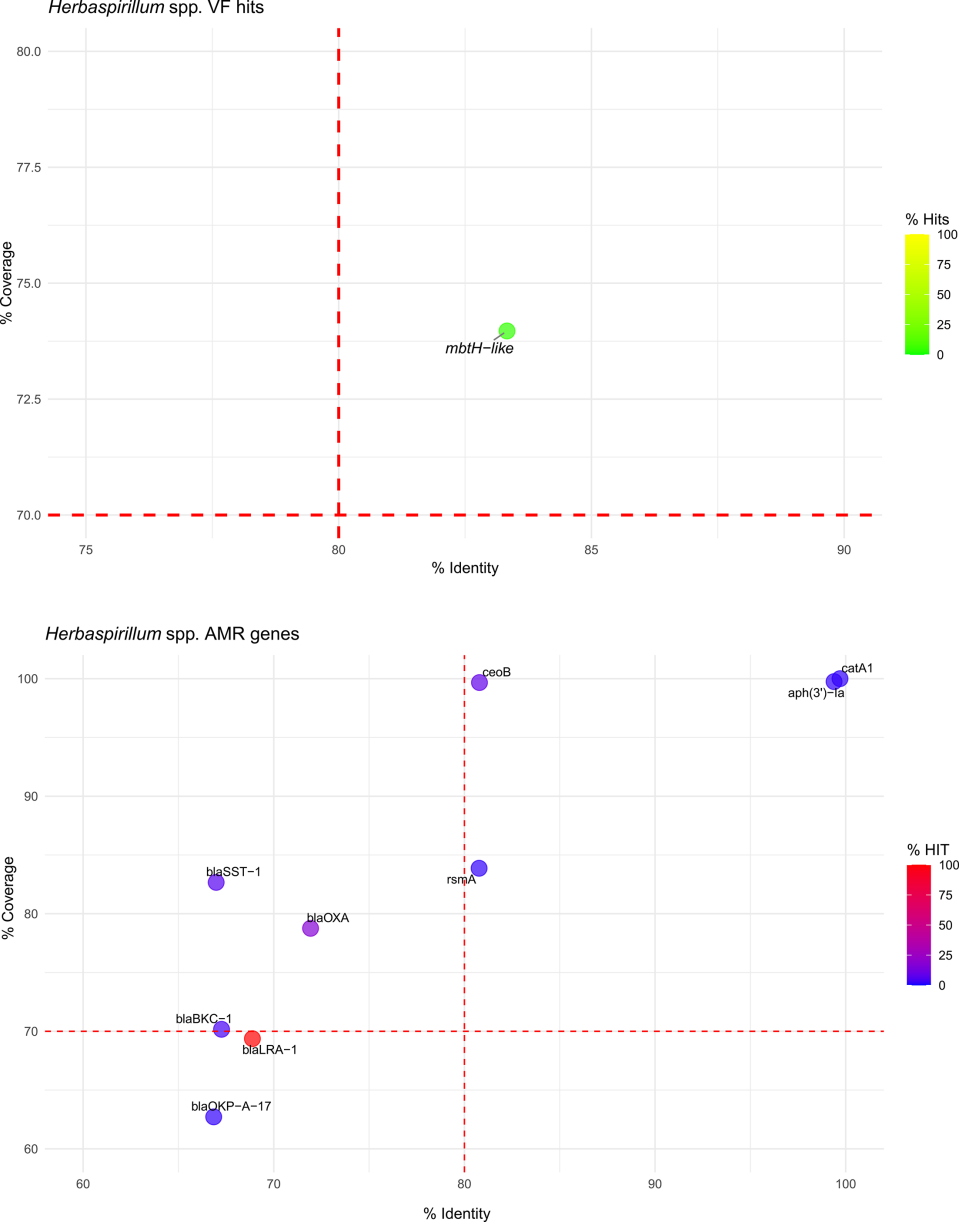
Bubble plot representing the VF (top) and AMR (bottom) hits in *Herbaspirillum* spp. The *X*-axis indicates the percentage identity, and the *Y*-axis shows the percentage coverage. Bubbles are coloured along a red-to-blue or yellow-to-green gradient based on the percentage of hits for AMRs or VFs, respectively. Red dashed lines represent the recommended thresholds at 80% identity and 70% coverage.

For *Herbaspirillum* spp., only one VF hit was detected among 46 genomes, corresponding to an MbtH-like protein from the pyoverdine cluster, found in two *Herbaspirillum rubrisubalbicans* strains DSM11543 and NBRC102523 (GCA_003719195.1 and GCA_001591225.1). In terms of AMR genes, the number of hits was also limited, with *ceoB* identified in 8% of genomes which can confer resistance to chloramphenicol antibiotics, with no other significant resistance genes detected (Fig. 5 and Table S3).

The overall number of hits for both AMR and VFs in the analysed *Arthrobacter* genomes was very limited (Table S3). The most common VF detected was the isocitrate lyase (*icl*) gene, found in four genomes: *Arthrobacter ginkgonis* strain JCM 30742 (GCA_039540195.1), *Arthrobacter hankyongi* strain I2-34 (GCA_022012395.1) and *Arthrobacter sulfonylureivorans* strains LAM7117 and S4-C24 (GCA_004000035.1 and GCA_022637435.1). Other VF hits were associated with cell motility (Table S3). Regarding AMR genes, a *bla_TEM_*_-116_ gene was detected in *Arthrobacter cavernae* strain PO-11 (GCA_017368795.1), while four hits of the *cmx* gene, which confers resistance to chloramphenicol, were identified in four *Arthrobacter koreensis* strains Gar NS 3, NPDC056645, NPDC058034 and NPDC058028 (GCA_035792235.1, GCA_042792195.1, GCA_042885795.1 and GCA_042885935.1). Additionally, the *aph*(3″)-Ib and *aph*(6)-Id genes, both associated with streptomycin resistance, were found in *Arthrobacter stackebrandtii* strains CCM 2783 and DSM 16005 (GCA_017876675.1 and GCA_003217635.1).

### *β*-lactam resistance genes, conserved regions and IS

The bioinformatic analysis initially set the thresholds at 80% identity and 70% coverage to capture only the most relevant AMR and VF hits, as supported by EFSA [38]. However, the ELS revealed that even if not detected with default parameters, *β*-lactamase genes were expected in *Azospirillum* [47]. To resolve this, thresholds were lowered to 60% identity and 60% coverage, allowing the identification of genes with sequence variations that could possibly confer resistance or virulence, even if deviating from the well-characterized reference sequences deposited on CARD or ResFinder. The comprehensive list of putative *β*-lactamases is presented in Table S4.

This approach revealed six distinct *β*-lactamase-like genes in 88% of the *Azospirillum* genomes analysed (Fig. 4), five *β*-lactamase-like genes in 100% of the *Herbaspirillum* genomes (Fig. 5) and none in *Azotobacter*. While these hits were initially undetected, mostly due to lower sequence identity compared to *β*-lactamase genes catalogued in the databases CARD and ResFinder, the adjusted thresholds allowed the detection of genes with partial similarity to known *β*-lactamase. The partial hit does not indicate these features as clear *β*-lactamases, but annotation of those genomes on NCBI confirms those as putative copies of *β*-lactamase genes (class A *bla* or class D *bla_OXA_*) in nearly all *Azospirillum* and *Herbaspirillum* genomes. For *Azospirillum* spp., only one copy of these genes was found annotated in each genome, with the exceptions of *Azospirillum fermentarium* CC-LY743 (GCA_025961205.1), which carries both *bla* and *bla_OXA_* genes, and *Azospirillum thiophilum* JJ-ME9A6 (GCA_943371305.1) and *Azospirillum griseum* L-25–5 w-1 (GCA_003966125.1), which lacked annotated *β*-lactamase genes entirely. Similarly, in *Herbaspirillum*, sequences with similarity to *β*-lactamase-like sequences were detected in all genomes except for *H. seropedicae* strain SMAGNO95 (GCA_039594535.1). Consistently, the closest hit on the database observed in both *Azospirillum* and *Herbaspirillum* genomes for these sequences was *bla_LRA_*_-1_ (accession number ARO:3002482), a known *β*-lactamase identified in Alaskan soil through functional metagenomics [42] found in 79% of *Azospirillum* and 100% of *Herbaspirillum* genomes.

**Fig. 6.**
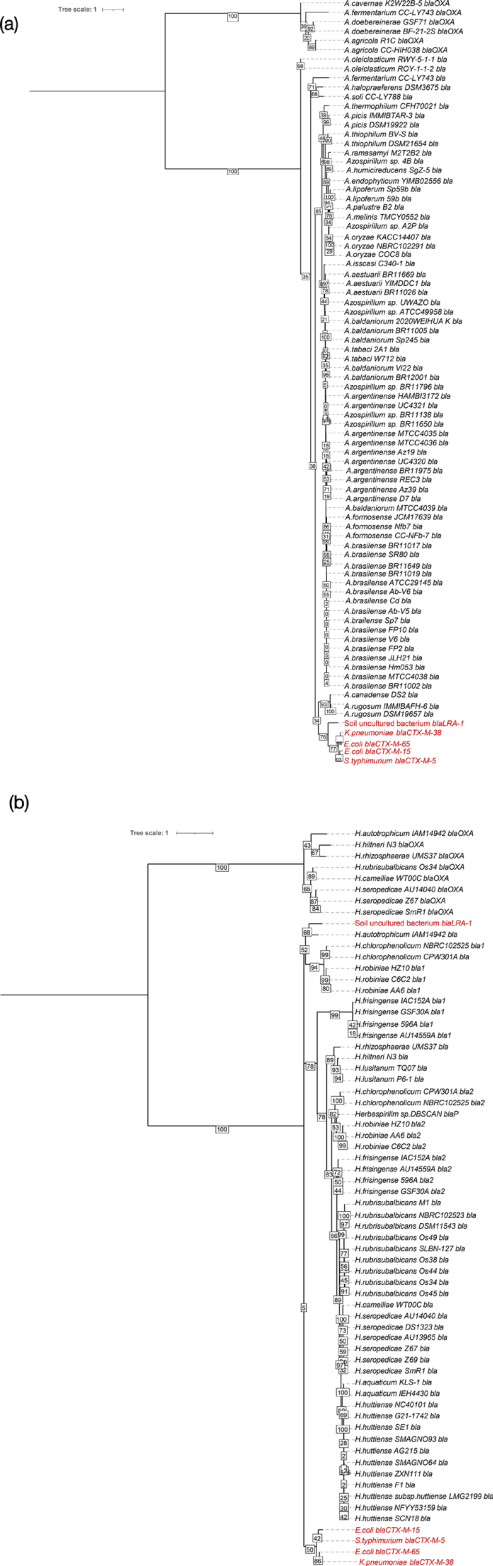
*β*-lactamase genes phylogenetic tree of sequences identified in (a) *Azospirillum* and (b) *Herbaspirillum* genomes. Clinically relevant *β*-lactamases are included in red for comparison. The branch lengths measure similarity differences in *bla* sequences at the amino acid level. Numbers reported at the nodes are 1–100 bootstrap values.

Moreover, the genetic context surrounding these genes is remarkably conserved in both genera. Specifically, when examining the regions adjacent to these putative *β*-lactamase genes, we consistently observed an upstream, divergently oriented *ampR* gene located upstream to the *β*-lactamase-like sequences (Fig. S1). This conserved gene structure is characteristic of operons involved in *β*-lactamase expression, suggesting that these genes may indeed confer *β*-lactamase activity, despite not matching known *β*-lactamase genes at higher identity thresholds. Nevertheless, clinically important CTX-M-type *β*-lactamases clustered separately on the phylogenetic tree, suggesting distinct evolutionary pathways of these *β*-lactamases (Fig. 6).

After conducting an ICEfinder analysis on complete *Azospirillum* and *Herbaspirillum* genomes, no integrative or conjugative elements were identified in any of the analysed non-QPS genomes, although some putative integrative and mobilizable elements (IMEs) and integrative and conjugative elements (ICEs) of T4SS type were located on some chromosomes (Table S5). Other mobile elements, like plasmids or transposons, can mediate gene transfer. The *bla* hits were found located on either the main chromosome or alternatively on smaller chromids but never on plasmids.

### Minimum inhibitory concentration

According to the types of hits that emerged during the bioinformatic analysis, the presence of AMR genes in *Azospirillum* genomes, particularly genes such as *bla*, *ceoB*, *catB*1 and *floR*, suggests potential resistance to *β*-lactams, represented by ampicillin, and amphenicols, namely chloramphenicol. To verify the phenotype associated with these findings, MIC tests were performed on four *Azospirillum* spp. strains according to the ISO 10932:2010 standards. The results, presented in Table 1, show that the *Azospirillum* strains tested share similar resistance profiles. The strains resulted to be sensitive to the lowest concentrations of gentamicin (GEN) ranging from 0.5 to 1 µg ml^−1^ and kanamycin (KAN) at 2 µg ml^−1^. The strains also demonstrated to be susceptible to streptomycin (STR), showing inhibitory activity at concentrations ranging from 1 to 4 µg ml^−1^, with the exception of *As. brasilense* Sp7, which was inhibited by 32 µg ml^−1^ of streptomycin. Furthermore, the strains were inhibited by 1 µg ml^−1^ of tetracycline (TET) and 16 µg ml^−1^ of chloramphenicol (CHL). Finally, the *Azospirillum* strains were found to be completely resistant to clindamycin (CLN) and ampicillin (AMP), with MIC values exceeding the resistance thresholds.

**Table 1.** MIC values (µg ml^−1^) detected in four strains belonging to the genus *Azospirillum* following the ISO 10932:2010 standards

Antibiotic (µg ml^−1^)	*AMR gene*	*As. brasilense* Sp 7	*As. argentinense* UC4321	*As. argentinense* UC4322	*Azospirillum* sp. UWAZO
Gentamicin	nd	1	1	0.5	0.5
Kanamycin	nd	2	2	2	2
Streptomycin	nd	32	4	1	1
Tetracycline	nd	1	1	1	1
Erythromycin	nd	>8	8	8	8
Clindamycin	nd	>16	>16	>16	>16
Chloramphenicol	*catB*1	16	16	16	16
Ampicillin	*bla_LRA_*_-1_	>16	>16	>16	>16

## Discussion

In this study, we employed a WGS approach integrated with ELS, adapting the QPS framework to microbial taxa already used as biostimulants, to gain insights into their safety. This combined methodology led to a thorough characterization of both QPS and non-QPS biostimulants, specifically *B. velezensis*, *P. megaterium*, *R. palustris*, *Arthrobacter* spp., *Azotobacter* spp., *Azospirillum* spp. and *Herbaspirillum* spp. Through this approach, we have examined their taxonomic classifications and identified AMR and VF genes for the evaluation of their safety profiles.

Although most genomes clustered as expected after species reassignment based on ANI values, using the standard threshold of 95% ANI, a subset of *Azospirillum* genomes exhibited inconsistencies between their ANI results and phylogenetic placement. Specifically, strains *As. argentinense* UC4321 and HAMBI3172 and *As. baldaniorum* MTCC4039 and 2020WEIHUAK showed ANI values slightly exceeding the 95% threshold with the reassigned type strain (Table S1). Despite these ANI values above the threshold for species demarcation, the phylogeny-based analysis (Fig. 2b) places them separately, indicating some genetic distinctions. This discrepancy suggests that the standard ANI cutoff of 95% may not be stringent enough for precise species delineation within the *Azospirillum* genus. Previous studies have proposed that a higher ANI threshold, such as 96%, may be more appropriate for certain bacterial groups where genomic diversity is higher [48]. The inconsistency between ANI-based classification and phylogenetic placement highlights limitations in current classification methods for *Azospirillum* and underscores the need for taxonomic refinement. A significant number of *Arthrobacter* genomes were reclassified as *Arthrobacter* sp. due to their inability to match any known type strains. This observation highlights the taxonomic complexity within the genus. One contributing factor is the high genomic diversity and restricted core genome of *Arthrobacter* species, which complicates the correlation between genomic data and taxonomic classification [49]. Accurate taxonomic classification is crucial for safety assessments, as it allows for reliable predictions of gene content and potential functional traits, including AMR and VF genes [50]. Therefore, ensuring that strains of interest are correctly classified and do not belong to clusters with known safety concerns is fundamental for a thorough risk assessment [51]. In fact, if the strains of interest fall in a taxonomic unit regarded as QPS, the strain would benefit from a quicker evaluation requiring fewer safety aspects, thus being more cost-effective. Safety assessments derived from the genome-wide analysis of AMRs and VFs indicate that neither the QPS nor the non-QPS strains studied are likely to introduce significant known risk factors into agricultural environments. As anticipated for QPS taxa, the genome-wide analysis showed minimal VFs and AMRs presence. No VFs were identified in *R. palustris* and *P. megaterium*, whereas only a single VF related to phenazine biosynthesis (*phzA*1) was detected in * B. velezensis* strain A6 (Table S3). Although phenazine biosynthesis is usually known to play a role in biocontrol activity, it may also be associated with cytotoxicity [52].

The AMR findings in *B. velezensis* largely confirm previous EFSA assessments [38]. Both studies highlight the widespread presence of *tetL* and *clbA*, which confer resistance to tetracycline and multiple antibiotic classes, respectively [53]. Notably, *tetL* and *clbA* are constitutive or intrinsic AMR genes and are generally not considered a risk, as they typically do not undergo HGT events [54]. However, our study did not detect either *lmrB*, which confers resistance to lincomycin, or *rphC*, which provides resistance to rifamycin, both identified in EFSA’s analysis. In *P. megaterium* genomes, the sole AMR detected was *lsaB*, identified in only 25% of the genomes. This gene encodes an ABC-F-type ribosomal protection protein known to confer resistance to clindamycin, aligning with findings in other *Priestia* species [55]. In *R. palustris* genomes, the only AMR gene detected was *aph*(3′)-IIa, which encodes an aminoglycoside phosphotransferase that confers resistance to aminoglycoside antibiotics by phosphorylating and inactivating them. These findings indicate that *R. palustris*, *B. velezensis* and *P. megaterium*, as QPS taxa, possess limited AMR profiles primarily involving intrinsic resistance mechanisms, posing minimal concern for virulence or AMR dissemination. Overall, our findings confirm the low pathogenicity potential of these species and suggest a minimal contribution to the spread of AMRs, supporting their classification as QPS [38].

For non-QPS taxa, VF hits were minimal in *Arthrobacter*, *Herbaspirillum* and *Azospirillum* genomes (Figs4  5), while *Azotobacter* displayed a broader range (Fig. 3). Detected VFs included genes related to alginate production (*algA*, *alg8*, *algC*), type IV pili formation (*pil*), LPS synthesis (*waaA*, *waaF*, *waaG*, *waaP*), T6SS (*hsiB*1, *hsiC*1, *hsiF*1) and isocitrate lyase (*icl*), the latter found in four *Arthrobacter* genomes. These traits, although present in opportunistic pathogens, primarily serve adaptive roles in environmental bacteria, enabling survival, competition and host interactions in challenging habitats like soil and the rhizosphere.

In pathogenic contexts, these genes can function as VFs. For example, in *Pseudomonas aeruginosa*, alginate production is essential for biofilm formation, allowing the bacterium to evade the immune system and persist in chronic infections [56]. Likewise, *P. aeruginosa* utilizes the T6SS to inject toxic effectors into host cells and competing bacteria, enhancing its virulence [46,57]. However, in plant-associated bacteria, the same genes contribute to beneficial functions. Type IV pili, alginate production genes and T6SS components facilitate root colonization, adhesion, biofilm formation and host recognition, improving rhizosphere adaptation [58,61]. Similarly, *icl* provides metabolic flexibility and stress tolerance, aiding survival in nutrient-poor environments, while LPS biosynthesis genes act as microbe-associated molecular patterns that trigger plant immune responses and can support biocontrol potential [62]. These VFs may also facilitate metabolite exchange between rhizobacteria and host plants, strengthening mutualistic interactions [63]. While the presence of these traits in opportunistic pathogens raises regulatory considerations, it is crucial to note that such genes alone do not define a pathogen. In highly competitive environments like soil, bacteria often retain these genes as part of their adaptive toolkit, enhancing rhizocompetence and enabling them to outcompete other microbes [58,64]. Nonetheless, their detection highlights the importance of thorough evaluation to distinguish adaptive survival mechanisms from potential risk factors in non-QPS strains.

Regarding AMR genes in non-QPS taxa, in the *Arthrobacter* genomes, the overall number of detected AMR genes was very limited, underscoring the generally low prevalence of these traits within the genus (Table S3). Notably, the detection of a single broad-spectrum *β*-lactamase gene (*bla_TEM_*_-116_) in the *A. cavernae* strain PO-11 suggests that *Arthrobacter* has the potential to harbour clinically relevant AMR genes, possibly acquired through HGT. However, considering the large dataset of 190 genomes, the very few AMR and VF hits highlight the low potential for pathogenicity and AMR dissemination within *Arthrobacter* spp. These findings align with the genus’s established reputation as a group of environmental bacteria primarily involved in soil nutrient cycling rather than opportunistic infections [65,66]. *Azotobacter* genomes exhibited some AMR hits, primarily involving efflux pump-related genes targeting fluoroquinolones, such as *mexB*, *mexE* and *mexF*, found in a notable proportion of genomes (Fig. 3). These genes, commonly associated with intrinsic resistance mechanisms, were previously described by Horna *et al*. [67] as typical efflux pumps of the soil resistome. The AMR profile of *Azospirillum* included *ceoB* (22%), *catB*1 (10%) and *floR* (4%), all associated with chloramphenicol resistance (Fig. 4). Importantly, a single broad-spectrum *β*-lactamase gene *bla_TEM_*_-116_ was detected in *As. brasilense* strain Ab-V6, indicating that also *Azospirillum* can acquire clinically relevant AMRs. Nevertheless, *As. brasilense* Ab-V6 has been widely used to study plant–microbe interactions in several pot and field trials [68,69], which should have contributed to the spread of clinically relevant plasmid-encoded TEM-116. The limited occurrence of *bla_TEM_*_-116_ or any other clinically relevant AMR in *Azospirillum* genomes in this study suggests a low likelihood of gene transfer across the genus. In *Herbaspirillum*, AMR hits were limited to *catA*1 (2%), *ceoB* (9%) and *rsmA* (2%), found in a small portion of the genomes (Fig. 5). The minimal presence of VFs and the absence of widespread, clinically important AMRs across the genomes of *Azotobacter*, *Azospirillum* and *Herbaspirillum* support a low-risk profile for these genera in environmental applications.

The genome-wide research was carried out in parallel with ELS, which revealed that several *Azospirillum* species are naturally resistant to *β*-lactam antibiotics due to the production of *β*-lactamase enzymes [47,70, 71]. This resistance is well-documented but was not detected in our initial genome analysis using the recommended thresholds. Recognizing this discrepancy, we lowered the thresholds to 60% coverage and 60% identity to capture *β*-lactamase genes that may have significant sequence divergence from the reference genes in the databases. This adjustment led to the detection of multiple *β*-lactamase genes in a high proportion of *Azospirillum* genomes. According to the database, the most similar sequence found in 71% of the *Azospirillum* genomes was *bla_LRA_*_-1_ (accession number ARO:3002482), a gene with known *β*-lactamase activity identified in Alaskan soil and described in [42]. In the study, *bla_LRA_*_-1_ was confirmed to confer resistance to *β*-lactam antibiotics when cloned into *Escherichia coli* DH5*α*.

Although the hits found with genome-wide analysis were below the threshold of relevance and thus cannot be assigned with certainty to *bla_LRA_*_-1_, the widespread presence of *bla* genes, along with documented resistance in many strains, supports the hypothesis that *β*-lactam resistance in *Azospirillum* is intrinsic. Application of the same relaxed thresholds to *Herbaspirillum* genomes also resulted in the detection of *β*-lactamase genes, particularly *bla_LRA_*_-1_ in 100% of the genomes (Table S4 and Fig. 5), indicating multiple copies of *β*-lactamase genes in some genomes. The necessity to lower the thresholds underscores the limitations of relying solely on default parameters in bioinformatic analyses, especially when dealing with environmental micro-organisms that may harbour genes with significant sequence variability. This situation highlights the need for more comprehensive studies on soil micro-organisms to enhance the existing databases and expand our knowledge of AMR and VF genes in environmental microbes. Improving the characterization of these organisms will not only refine bioinformatic tools and databases but will also contribute to more accurate and reliable safety assessments throughout the food chain, starting from soil, which can be considered a pool of AMRs [72].

The *β*-lactamase-like genes found in *Azospirillum* and *Herbaspirillum* are also putatively annotated as *β*-lactamase on NCBI (functionality inferred through sequence similarity). These genes showed lower sequence identity to the reference genes in the databases, likely due to evolutionary divergence or the presence of novel variants not yet catalogued. This is confirmed by the phylogenetic tree (Fig. 6) which shows separate clustering branches not only from clinically relevant sequences but also from the original sequence of *bla_LRA_*_-1_, the most similar sequence deposited in the CARD database. To further confirm the *β*-lactamase function of these shared genetic features, we observed that the genetic regions surrounding these *β*-lactamase genes, once aligned, show similar structures upstream and downstream of the *bla* gene. This was true for most *Azospirillum* and *Herbaspirillum* genomes studied, with conserved regions near *β*-lactamase genes and similar patterns across all 78 genomes with few exceptions (Fig. S1). This provides particular confidence in the identification of these sequences as *β*-lactamases, as the presence of an upstream and divergently organized *ampR* gene was found either on the same or opposite strand of *bla*. Likewise, in *Herbaspirillum*, the same pattern of an *ampR* gene divergent to *β*-lactamase was observed, indicating a conserved genetic context surrounding these resistance elements for both genera. This reinforces the fact that soil is a reservoir of AMRs that are not well characterized and highlights the importance of customizing analysis parameters based on prior knowledge to ensure comprehensive detection of relevant genes.

Few studies have investigated the genetics involved in the *β*-lactamase activity of *Azospirillum* in depth. One study focused on the exact *bla* genes adjacent to *ampR* that emerged during our analysis and confirmed *β*-lactam resistance in the *As. baldaniorum* strain Sp245 [71]. This study demonstrated that these genes conferred resistance to several *β*-lactam antibiotics, including ampicillin, cephalexin, cefadroxil, cefazolin, cefoperazone, piperacillin and ticarcillin. Most other studies have been limited to phenotypic characterization. For instance, one study obtained the MIC of 63 strains of *Azospirillum*: gentamicin at 32 µg ml^−1^ inhibited the growth of all strains, as did streptomycin at 256 µg ml^−1^ and spectinomycin at 512 µg ml^−1^. Chloramphenicol and tetracycline inhibited the growth of 98.4% of the strains at concentrations of 8 µg ml^−1^, while all strains showed high-level resistance to trimethoprim (MIC≥1,024 µg ml^−1^) and *β*-lactam antibiotic [47]. These results about MIC values for ampicillin and chloramphenicol were in line with those obtained in our study, although different methodologies were adopted. For gentamicin, streptomycin and tetracycline, the MIC values found in our study were different. As there are no cutoff values for *Azospirillum* spp., it is not possible to truly compare the values obtained and establish a cutoff between resistance and susceptibility. MIC values≥16 µg ml^−1^ for ampicillin and chloramphenicol can be explained by the presence of the *bla_LRA_*_-1_ and *catB*1 genes present in the strains tested. For the other antimicrobials tested, it will be essential to determine the cutoff values first to correlate the values with the genetic context.

Overall, the insights gathered through the ELS suggest that *β*-lactam resistance in *Azospirillum*, and likely in *Herbaspirillum* as well, is intrinsic. Given that both genera are involved in plant–microbe interactions, it is reasonable to speculate that these resistance features may enhance their rhizocompetence, enabling them to outcompete other rhizobacteria and facilitate interactions. This finding suggests that the *β*-lactamase genes identified in *Azospirillum* and *Herbaspirillum* are unlikely to be easily transferred between bacteria through HGT.

Collectively, although our approach could not conclusively identify these *bla* genes as specific *β*-lactamases due to sequence variability and the lack of data in database repositories, we were able to infer their potential *β*-lactamase function based on ELS findings. This inference is supported by several factors: (i) nearly all genomes contained at least one copy of these genes, with few exceptions indicating that the presence of this gene is most likely due to vertical gene transfer; (ii) putative annotations in NCBI indicate the presence of *β*-lactamase genes in practically every genome; (iii) gene structure, including the adjacent *ampR* gene, was conserved across both genera. These findings highlight the importance of considering sequence variability and conserved genetic contexts in AMR gene identification, especially when dealing with environmental micro-organisms that may harbour novel resistance elements not captured by standard databases.

Finally, our study indicates that genomics data can be utilized in evaluating the risks associated with non-QPS plant growth-promoting rhizobacteria (PGPR) strains. By using WGS alongside ELS, we were able to effectively identify bacterial features crucial for safety assessment. Nonetheless, this method presented some constraints. A significant limitation was the variation and divergence of gene sequences in soil micro-organisms, which may not align well with existing reference genes in databases. This might result in the under-detection of certain AMR genes unless detection thresholds are modified, as observed with *β*-lactamase genes in *Azospirillum* and *Herbaspirillum*. Furthermore, the absence of extensive databases for environmental micro-organisms requires tailored bioinformatic analyses and thorough result interpretation. Beyond database limitations, establishing species-specific MIC thresholds for non-QPS MBs is essential for distinguishing intrinsic from acquired resistance and improving risk assessment. The current lack of both comprehensive databases and defined MIC thresholds remains the main limitation. Addressing these gaps would greatly enhance the reliability of non-QPS strain evaluations. Consequently, while genomics serves as a useful tool for evaluating the safety of non-QPS PGPR strains, it should be supplemented with phenotypic testing and a comprehensive evaluation of the scientific literature to address these limitations and guarantee an all-encompassing risk assessment.

## Supplementary material

10.1099/mgen.0.001391 Uncited Supplementary Material 1.

10.1099/mgen.0.001391 Uncited Supplementary Material 2.

## Abbreviations

AMR: antimicrobial resistance
ANI: average nucleotide identity
CARD: comprehensive antibiotic resistance database
CRISPR: clustered regularly interspaced short palindromic repeats
dDDH: digital DNA–DNA hybridization
EBIC: European Biostimulants Industry Council
EFSA: European Food Safety Authority
ELS: extensive literature search
HGT: horizontal gene transfer
ICEs: integrative and conjugative elements
ICEs: integrated conjugative elements
IME: integrative and mobilizable elements
IS: insertion sequences
iTOL: Interactive Tree of Life
LPS: lipopolysaccharide
MBs: microbial biostimulants
MGEs: mobile genetic elements
MICs: minimum inhibitory concentrations
NCBI: Nation Center for Biotechnology Information
PGPR: plant growth-promoting rhizobacteria
QPS: qualified presumption of safety
T6SS: type VI secretion system
VFs: virulence factors
WGS: whole genome sequencing
