# Supplementary material for: Comprehensive genome-wide analysis for the safety assessment of microbial biostimulants in agricultural applications
Source: Microb Genom. 2025 Apr 28;11(4):001391. doi: 10.1099/mgen.0.001391 (PMC12038027; doi:10.1099/mgen.0.001391)

Figure S1. Phylogenetic classification of the 189 *Arthrobacter* genomes analyzed based on their species-level phylogenetic relationship.

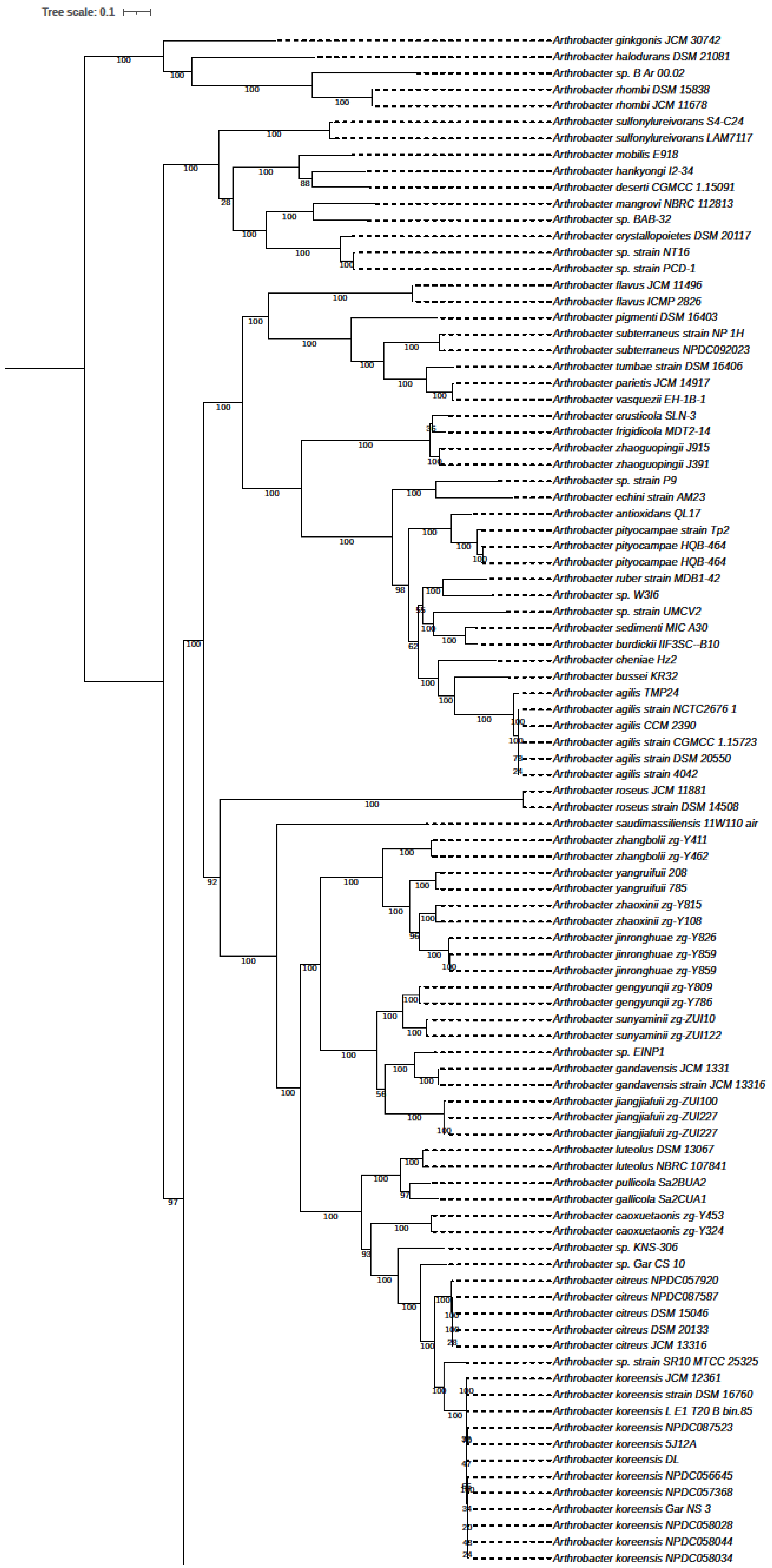

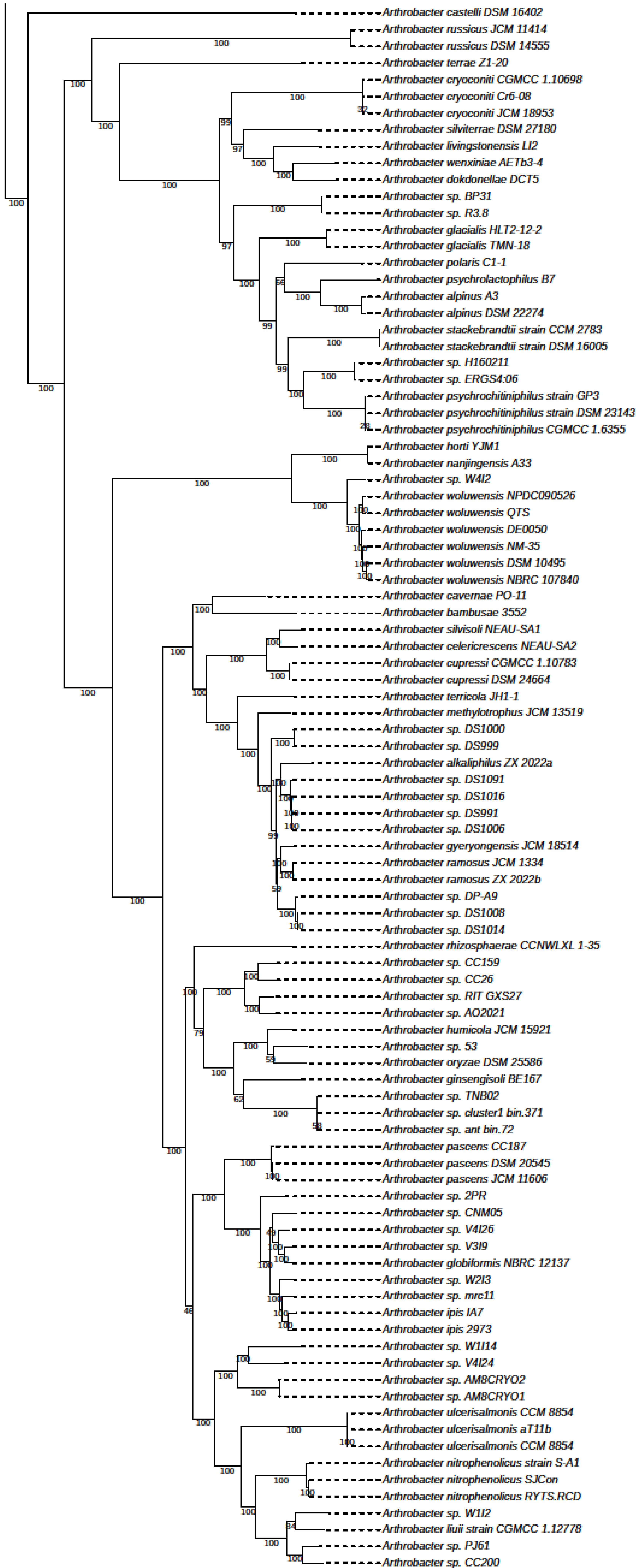

Arthrobacter castelli DSM 16402
Arthrobacter russicus JCM 11414
Arthrobacter russicus DSM 14555
Arthrobacter terrae Z1-20
Arthrobacter cryoconiti CGMCC 1.10698
Arthrobacter cryoconiti Cr6-08
Arthrobacter cryoconiti JCM 18953
Arthrobacter silviterrae DSM 27180
Arthrobacter livingstonensis LI2
Arthrobacter wenxiniae AETb3-4
Arthrobacter dokdonellae DCT5
Arthrobacter sp. BP31
Arthrobacter sp. R3.8
Arthrobacter glacialis HLT2-12-2
Arthrobacter glacialis TMN-18
Arthrobacter polaris C1-1
Arthrobacter psychrolactophilus B7
Arthrobacter alpinus A3
Arthrobacter alpinus DSM 22274
Arthrobacter stackebrandtii strain CCM 2783
Arthrobacter stackebrandtii strain DSM 16005
Arthrobacter sp. H160211
Arthrobacter sp. ERGS4:06
Arthrobacter psychrochitiniphilus strain GP3
Arthrobacter psychrochitiniphilus strain DSM 23143
Arthrobacter psychrochitiniphilus CGMCC 1.6355
Arthrobacter horti YJM1
Arthrobacter nanjingensis A33
Arthrobacter sp. W4I2
Arthrobacter woluwensis NPDC090526
Arthrobacter woluwensis QTS
Arthrobacter woluwensis DE0050
Arthrobacter woluwensis NM-35
Arthrobacter woluwensis DSM 10495
Arthrobacter woluwensis NBRC 107840
Arthrobacter cavernae PO-11
Arthrobacter bambusae 3552
Arthrobacter silvisoli NEAU-SA1
Arthrobacter celericrescens NEAU-SA2
Arthrobacter cupressi CGMCC 1.10783
Arthrobacter cupressi DSM 24664
Arthrobacter terricola JH1-1
Arthrobacter methylotrophus JCM 13519
Arthrobacter sp. DS1000
Arthrobacter sp. DS999
Arthrobacter alkaliphilus ZX 2022a
Arthrobacter sp. DS1091
Arthrobacter sp. DS1016
Arthrobacter sp. DS991
Arthrobacter sp. DS1006
Arthrobacter gyeryongensis JCM 18514
Arthrobacter ramosus JCM 1334
Arthrobacter ramosus ZX 2022b
Arthrobacter sp. DP-A9
Arthrobacter sp. DS1008
Arthrobacter sp. DS1014
Arthrobacter rhizosphaerae CCNWLXL 1-35
Arthrobacter sp. CC159
Arthrobacter sp. CC26
Arthrobacter sp. RIT GXS27
Arthrobacter sp. AO2021
Arthrobacter humicola JCM 15921
Arthrobacter sp. 53
Arthrobacter oryzae DSM 25586
Arthrobacter ginsengisoli BE167
Arthrobacter sp. TNB02
Arthrobacter sp. cluster1 bin.371
Arthrobacter sp. ant bin.72
Arthrobacter pascens CC187
Arthrobacter pascens DSM 20545
Arthrobacter pascens JCM 11606
Arthrobacter sp. 2PR
Arthrobacter sp. CNM05
Arthrobacter sp. V4I26
Arthrobacter sp. V3I9
Arthrobacter globiformis NBRC 12137
Arthrobacter sp. W2I3
Arthrobacter sp. mrc11
Arthrobacter ipis IA7
Arthrobacter ipis 2973
Arthrobacter sp. W1I14
Arthrobacter sp. V4I24
Arthrobacter sp. AM8CRYO2
Arthrobacter sp. AM8CRYO1
Arthrobacter ulcerisalmonis CCM 8854
Arthrobacter ulcerisalmonis aT11b
Arthrobacter ulcerisalmonis CCM 8854
Arthrobacter nitrophenolicus strain S-A1
Arthrobacter nitrophenolicus SJCon
Arthrobacter nitrophenolicus RYTS.RCD
Arthrobacter sp. W1I2
Arthrobacter liuii strain CGMCC 1.12778
Arthrobacter sp. PJ61
Arthrobacter sp. CC200

Figure S2. Comparison of the region flanking the *bla* genes (red boxes) across the 78 *Azospirillum* and 46 *Herbaspirillum* genomes. The divergently organized *ampR* gene is indicated as orange and blue boxes in *Azospirillum* and *Herbaspirillum*, respectively.

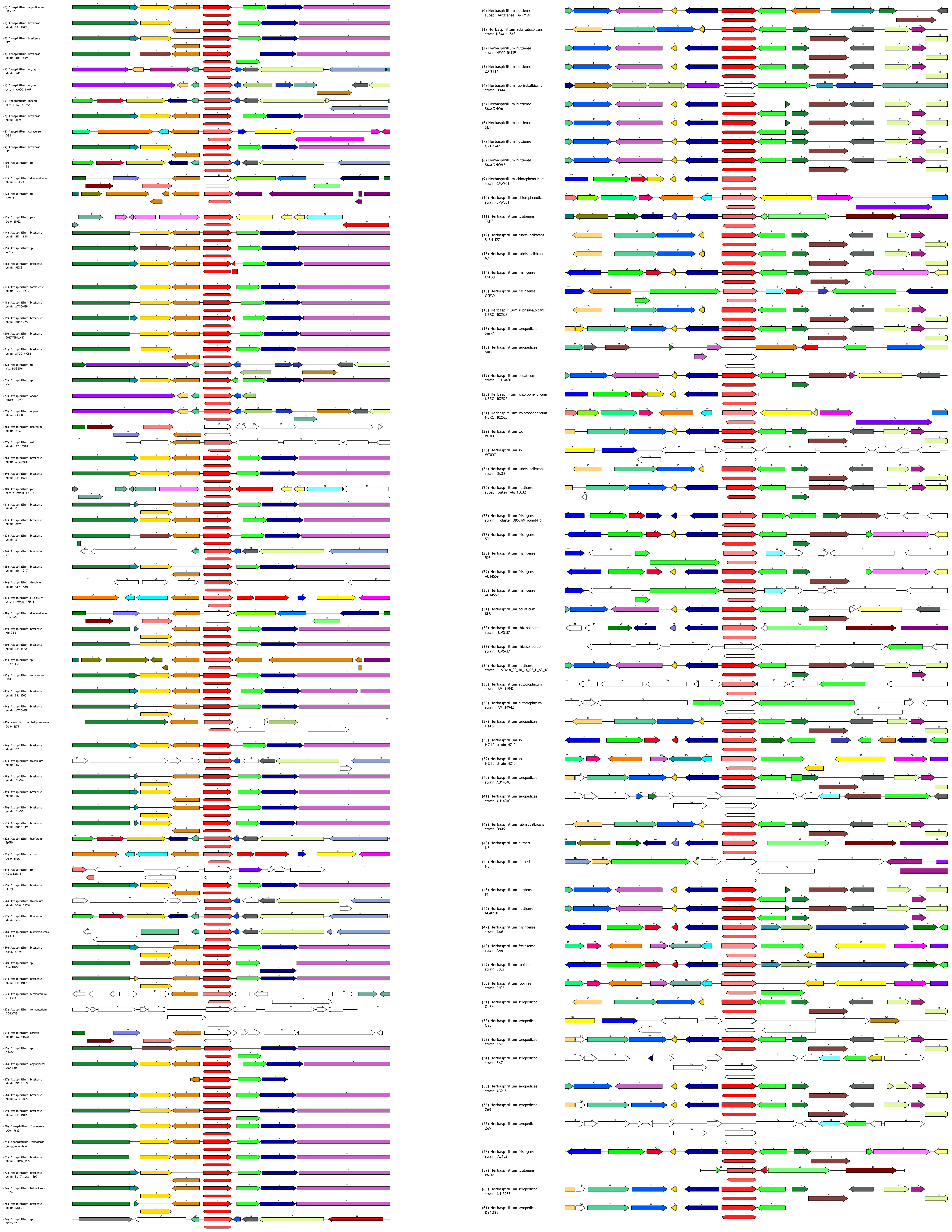

Supplement: Uncited Supplementary Material 1. [file mgen-11-01391-s001.pdf]
